# Supplementary figures and images for: The Gene Expression Profile Differs in Growth Phases of the Bifidobacterium Longum Culture
Source: Microorganisms. 2022 Aug 21;10(8):1683. doi: 10.3390/microorganisms10081683 (PMC9415070; doi:10.3390/microorganisms10081683)

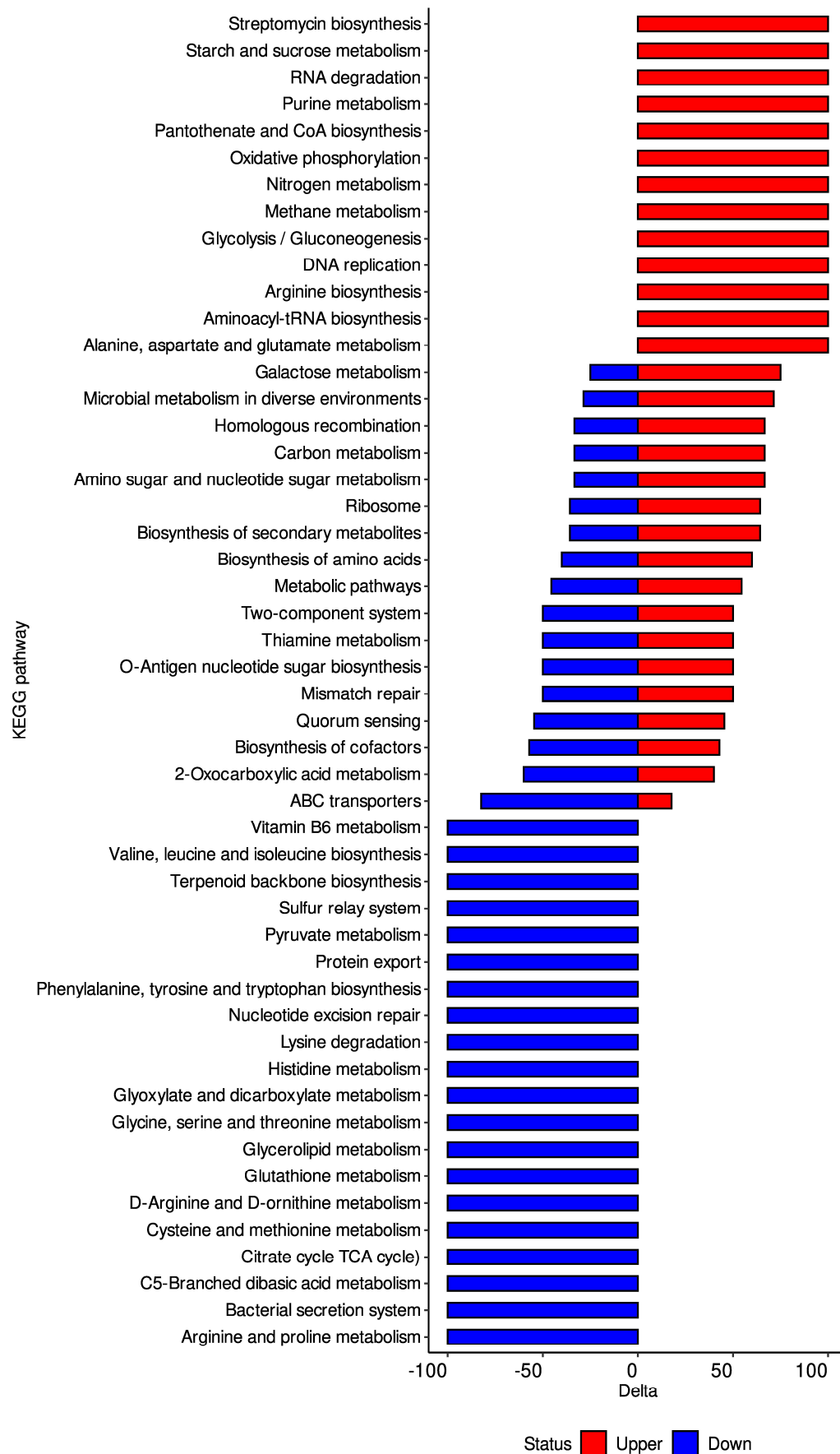

Figure S1: KEGG\_pathway (GT15\_ex compared to GT15\_lag)

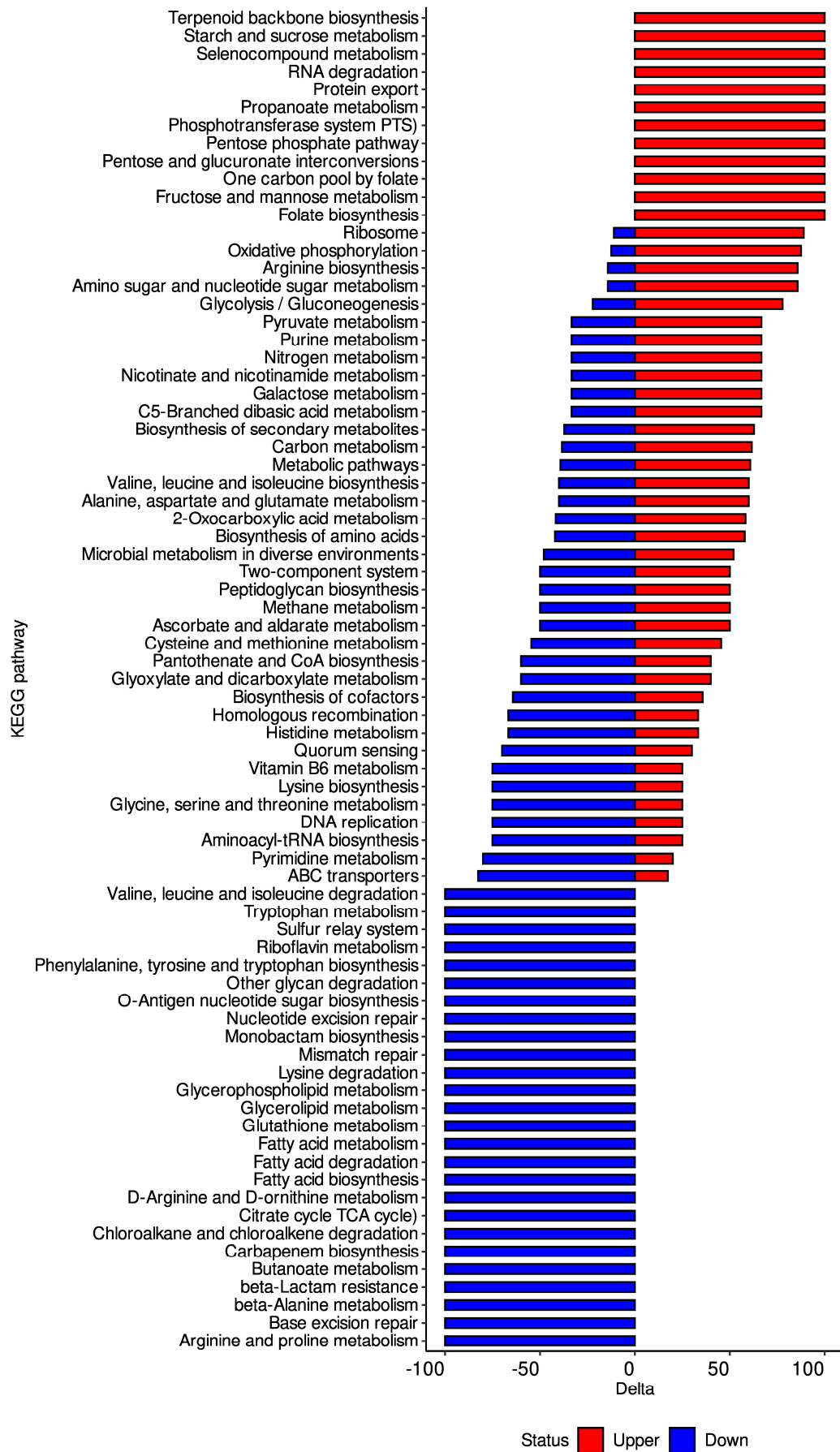

Figure S2: KEGG\_pathway (GT15\_st compared to GT15\_lag)

Supplement: Supplementary file 1 [file microorganisms-10-01683-s001.zip › Figures S1 and S2.pdf]
